# Global Ecological Pattern of Ammonia-Oxidizing Archaea

**DOI:** 10.1371/journal.pone.0052853

**Published:** 2013-02-28

**Authors:** Huiluo Cao, Jean-Christophe Auguet, Ji-Dong Gu

**Affiliations:** 1 Laboratory of Environmental Microbiology and Toxicology, School of Biological Sciences, The University of Hong Kong, Hong Kong, China; 2 Equipe Environnement et Microbiologie, UMR CNRS-IPREM 5254, Université de Pau et des Pays de l'Adour, Pau, France; 3 The Swire Institute of Marine Science, The University of Hong Kong, Hong Kong, China; Uppsala University, Sweden

## Abstract

**Background:**

The global distribution of ammonia-oxidizing archaea (AOA), which play a pivotal role in the nitrification process, has been confirmed through numerous ecological studies. Though newly available *amoA* (ammonia monooxygenase subunit A) gene sequences from new environments are accumulating rapidly in public repositories, a lack of information on the ecological and evolutionary factors shaping community assembly of AOA on the global scale is apparent.

**Methodology and Results:**

We conducted a meta-analysis on uncultured AOA using over ca. 6,200 archaeal *amoA* gene sequences, so as to reveal their community distribution patterns along a wide spectrum of physicochemical conditions and habitat types. The sequences were dereplicated at 95% identity level resulting in a dataset containing 1,476 archaeal *amoA* gene sequences from eight habitat types: namely soil, freshwater, freshwater sediment, estuarine sediment, marine water, marine sediment, geothermal system, and symbiosis. The updated comprehensive *amoA* phylogeny was composed of three major monophyletic clusters (i.e. *Nitrosopumilus*, *Nitrosotalea*, *Nitrosocaldus*) and a non-monophyletic cluster constituted mostly by soil and sediment sequences that we named *Nitrososphaera*. Diversity measurements indicated that marine and estuarine sediments as well as symbionts might be the largest reservoirs of AOA diversity. Phylogenetic analyses were further carried out using macroevolutionary analyses to explore the diversification pattern and rates of nitrifying archaea. In contrast to other habitats that displayed constant diversification rates, marine planktonic AOA interestingly exhibit a very recent and accelerating diversification rate congruent with the lowest phylogenetic diversity observed in their habitats. This result suggested the existence of AOA communities with different evolutionary history in the different habitats.

**Conclusion and Significance:**

Based on an up-to-date *amoA* phylogeny, this analysis provided insights into the possible evolutionary mechanisms and environmental parameters that shape AOA community assembly at global scale.

## Introduction

For the cycling of nitrogen on earth, a number of critical processes carried out by microorganisms have been recognized, including dinitrogen (N_2_) fixation, ammonification, nitrification, denitrification, and anammox (anaerobic ammonium oxidation) [1], [2]. Nitrification is defined as the oxidation of ammonia to nitrate through nitrite as an intermediate. The first step of nitrification, ammonia oxidation, is the rate-limiting one in the nitrification process [3]. For more than one hundred years, ammonia-oxidizing bacteria (AOB) from the beta- and gamma-proteobacteria class have been known to be the only organisms responsible for this biochemical step [4]. However, metagenomics radically changed the general perception of the nitrification process in unraveling the widespread Archaea of the Thaumarchaeota phylum as potential contributor to nitrification [5], [6]. This was later confirmed by the successful isolation of *N. maritimus* in pure culture [7].

More enrichments and isolates of AOA have been obtained subsequently [7]–[14], confirming the presence of putative ammonia monooxygenase subunits (i.e. *amoA*, a*moB* and *amoC*) within the genomes of Thaumarchaeota. Among these sub-units, the *amoA* gene has already been widely used as a reliable genetic marker to explore the diversity and abundance of AOA in various ecosystems [15], [16]. The study of AOA distribution patterns has advanced our understanding of the relationships between microbial community ecology and the environmental parameters driving their composition and abundance at local and regional scale. Wessen et al. (2011) reported large differences in AOA abundance in relation to pH variations over 107 sampling sites covering an area of 31500 km^2^ [17]. In addition, environmental parameters, such as pH, depth, nutrients and dissolved oxygen, were identified as potential factors determining the dominant phylotypes of the ammonia oxidizers and their diversity in ecosystems [18]. However, how ecological and evolutionary factors shape the community assembly of AOA on a global scale and whether it is possible to assign specific *amoA* lineages to each type of habitat, i.e. soil, freshwater, marine sediment, etc are still unanswered questions. Solving this matter would shed some light on the environmental and historical forces influencing AOA community distribution, diversity and ecology. Meta-analyses have proven to be useful approaches providing phylogeographical clues on key evolutionary and ecological aspects of bacteria [19], archaea [20] and denitrifiers [21]. Recently, two studies have demonstrated the prevalence of niche-based mechanism of community assembly over neutral processes for AOA at the global scale [22], [23]. Focusing on aquatic habitats, Biller and colleagues proposed salinity, water column depth, and temperature as potential sources of selective pressures driving the partitioning of AOA communities [23]. Comparing AOA and AOB, Fernandez and Casamayor observed larger phylogenetic richness and higher diversification rates in AOA than AOB [22].

In the present study, we investigate the underlying processes influencing AOA community distribution patterns using *amoA* sequences available from public repositories, similar with two previous studies. Although our analysis considered all natural habitats, a special emphasis on *amoA* sequences originating from estuarine and freshwater systems was made and this is different from two previous studies on comparison between AOA and AOB and aquatic habitats of AOA. In an attempt to understand the role of evolutionary processes involved in the observed community pattern, we calculated AOA diversification rates and pattern in each habitat and discovered that particularly low diversity of AOA in the marine habitat may be related to a more recent and accelerating diversification of AOA in this ecosystem.

## Results and Discussion

### Topology of the amoA phylogenetic tree

**Figure 1:**
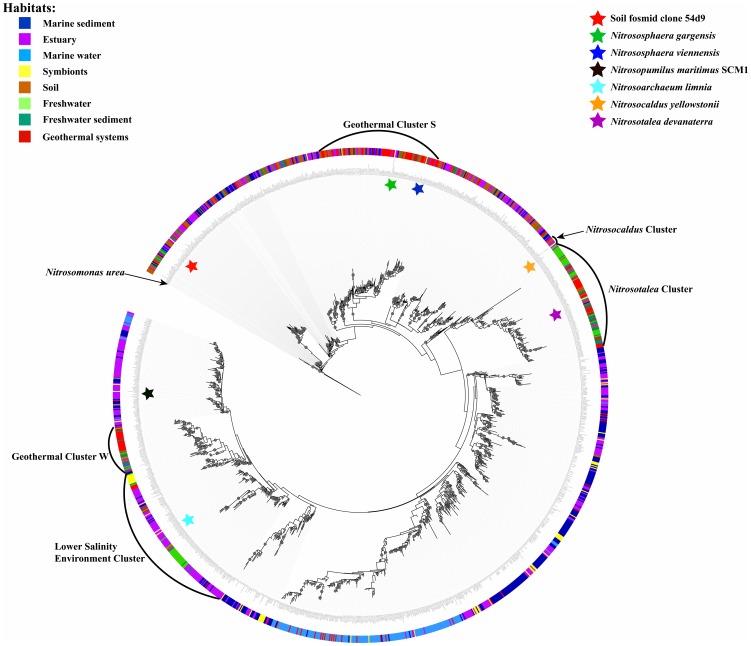
Phylogenetic tree based on archaeal *amoA* gene sequences from the variable samples on the global level using the maximum likelihood (ML) criterion. The credible support over 70% for each node was indicated with round circle on the node. The outer color circle around the phylogenetic tree suggested the different habitats.

Our *amoA* gene phylogenetic tree was composed of three major monophyletic clusters (i.e. *Nitrosopumilus*, *Nitrosotalea*, *Nitrosocaldus*) and a non-monophyletic cluster constituted mostly by soil and sediment sequences that we named *Nitrososphaera* (Fig. 1) following the nomenclature of Pester et al. (2012). Unlike the latter phylogeny, the *Nitrososphaera* cluster was located at the base of the tree and was not a monophyletic sister cluster of the *Nitrosocaldus* and *Nitrosopumilus*/*Nitrosotalea* clusters. The discrepancies between both phylogenies may be explained by the fact that Pester's phylogeny is based on sequences available publically in 2010. Since then, a significant amount of new sequences have been made available in the public databases and particularly from low salinity or freshwater habitats like estuarine and freshwater systems [11], [24], [25]. Although freshwater habitats have recently been proposed as one of the largest reservoirs of archaeal genetic diversity up to date [20], [26], only a few freshwater planktonic habitats have been surveyed for *amoA* gene diversity. These include rivers [25], oligotrophic lakes [24], [27], groundwater [28] and drinking water [29]. These studies on freshwater environments provided some new archaeal ammonia oxidizer lineages [24], [25], [27], indicating that planktonic freshwater habitats harbor typical *amoA*-containing ecotypes different from those found in soils and oceans [24]. Because these freshwater sequences were also clustered with those from acidic soils, e.g. the enrichment of *Candidatus* Nitrosotalea devanaterra, this cluster was named as *Nitrosotalea* cluster in agreement with Pester et al (Fig. 1) [30]. Freshwater sequences and those from low salinity environments, i.e. estuaries [31]–[33] and hot springs [8], [9], [34]–[37] formed another non-monophyletic group of several secondary clusters within the *Nitrosopumilus* cluster. This group of clusters, containing the low salinity archaeal *Nitrosoarchaeum limnia*, is separated clearly from other saline clusters and is temporally named as Low Salinity Environment Cluster (Fig. 1).

In contrast to *amoA* sequences from each habitat that tended to group in specific clusters, estuarine sequences were widely distributed across the phylogenetic tree (Fig. 1). This over-dispersion of estuarine sequences in the phylogenetic tree may be explained by the influence of freshwater discharge, soil drainage waters and coastal marine water intrusions in estuarine habitats. Hence, the ubiquitous distribution of estuarine sequences in the three major *amoA* phylogenetic clusters (i.e. *Nitrosopumilus*, *Nitrosotalea and Nitrososphaera clusters*) was not surprising.

### Phylogenetic ecology

**Figure 2:**
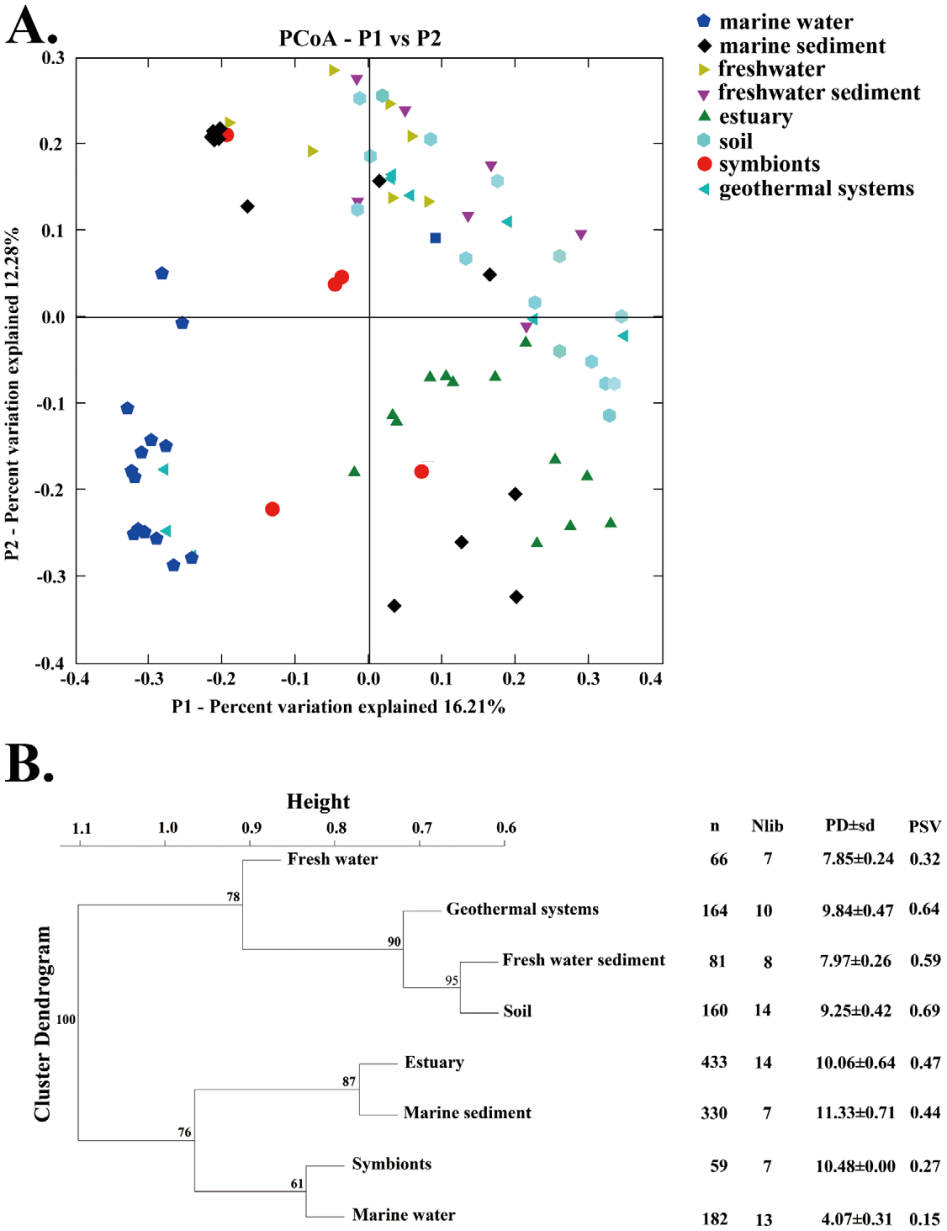
Principal coordinate analysis (PCoA) plot for archaeal *amoA* gene assemblages based on the eight types of habitats deduced from the online Fast UniFrac software (a). Hierarchical clustering analysis (UPGMA algorithm with 100 replicates Jackknife supporting test) for the all archaeal *amoA* gene sequences represent of eight types of habitats according to the online Fast UniFrac software. The number of sequence (n), number of libraries (Nlib), phylogenetic diversity with s.d. (PD±s.d.) and phylogenetic species variability (PSV) in each habitat is given. S.d. for PSV index was less than 0.001 for all habitats (b).

**Figure 3:**
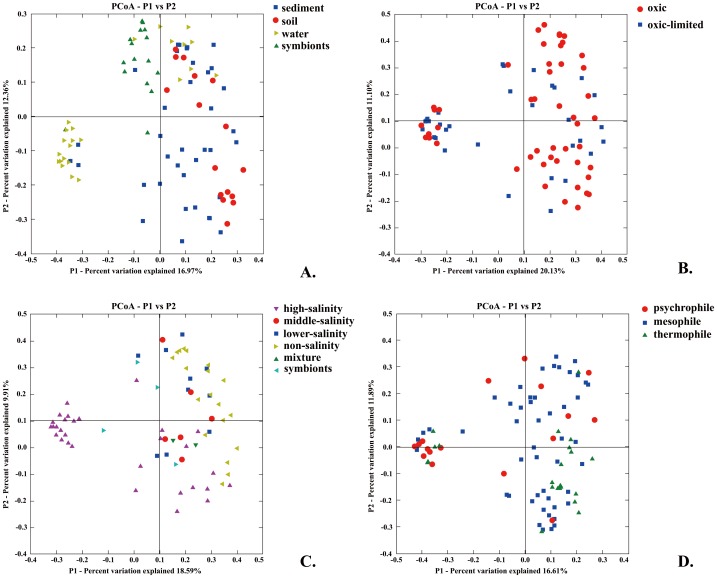
PCoA analyses for archaeal *amoA* gene assemblages based on the different environmental factors calculated by the online Fast UniFrac software (A, living style; B, oxygen; C, salinity; D, temperature).

The 85 environmental AOA clone libraries analyzed in this study were sorted into a principal coordinate analysis (PCoA) plot according to phylogenetic community similarity (Fig. 2). In agreement with the concept of habitat filtering [38] and as observed in previous studies on ribosomal or functional genes [19]–[23], AOA communities were more similar within habitats than among habitats (*R^2^* = 0.16, *P* = 0.001) (Fig. 2). Among the environmental variables common to all studies (i.e. salinity, lifestyle, temperature and oxygenation), salinity was the strongest factor explaining AOA community structure patterns. Salinity alone accounted for 8.6% (*P* = 0.003) of the total variance from the Unifrac analysis (Fig. 3a) and clearly separated saline habitats from non-saline habitats in a hierarchical clustering analysis (Fig. 2b). Previous studies analyzing prokaryotic phylogenies based on ribosomal [19], [20] and functional genes [23] have also revealed a clear separation between freshwater and marine lineages, suggesting that, similar to eukaryotes, salinity represented one of the most important evolutionary barrier preventing frequent environmental transitions [39]. Jones et al. [21] showed that this evolutionary segregation also applies to the *nirS* and *nirK* denitrifying genes. However, due to marked incongruences between ribosomal and denitrifying gene phylogenies, they could not rule out an important effect of horizontal gene transfer (HGT). Unlike denitrifying genes, archaeal *amoA* phylogeny seemed to be largely congruent with the archaeal ribosomal phylogeny [24], [40], [41]. Therefore, our analysis suggested that salinity rather than HGT may have a more significantly influence on the evolution of AOA and may be one of the most important evolutionary factors for N transforming microorganisms.

Together with salinity, our analysis suggested that other environmental variables such as lifestyle (*R^2^* = 0.06, *P* = 0.001) and temperature (*R^2^* = 0.04, *P* = 0.01) represented significant driving forces of AOA distribution pattern at global scale (Figs. 3a and 3d). Temperature was recently recognized as a key factor influencing AOA diversity in aquatic ecosystems [23]. The formation of monophyletic clusters by *amoA* sequences exclusively from marine sediment (Msed) or marine water column (Mwc) habitats may reflect the adaptation to sessile or planktonic lifestyle (Fig. 1). Similarly, the formation of typical geothermal clusters may illustrate the strong selective pressures exerted by high temperature in geothermal habitat. Surprisingly, oxygenation was not a significant factor (*R^2^* = 0.01, *P* = 0.09), although oxygen could be an important factor shaping AOA community structure in natural environments [42], [43]. Oxygen is generally the electron acceptor for AOA but an alternative energy metabolism involving nitrous oxide combined to oxygen as potential electron acceptor has been proposed [42], [44]. This suggests that AOA could survive well in low oxygen conditions. In agreement, an unexpected compositional overlap between *amoA* sequences from distinct environments characterized by a large variability in oxygen contents has been observed in previous works [43], [45], indicating that similar AOA communities could survive a broad range of oxygen concentrations.

Unfortunately, most of studies lacked detailed information on factors known to have an impact on AOA community structure (i.e. sampling depth, N species concentration, organic carbon, pH, sulfide, and phosphate levels) at local scale could not be tested at global scale. Particularly, pH has been shown to affect soil microbial community structure in large-scale studies based on 16S rRNA gene [46], [47], denitrifying genes [48], [49] and *amoA* gene [30], [50], pH may therefore represent another important evolutionary force for N cycling microorganisms at global scale. However, to date, this parameter has been largely ignored in most planktonic or sediment AOA surveys.

### Diversity and Diversification

Rarefaction curves (Fig. S1), diversity indices (Fig. 2b) (i.e. phylogenetic diversity (PD) and phylogenetic species variability (PSV)) were determined for each habitat. As previously observed, highest PD values were found in marine sediments [22], [23]. Here we also observed high PD values in symbionts and estuarine sediments indicating that these habitats may be the largest reservoir of AOA diversity and, therefore, promising environment for the discovery of new AOA ecotypes. In agreement, the accumulation of OTU's in rarefaction curves did not reach an asymptote, evidencing that AOA diversity is far from exhaustively sampled. Estuarine sediment diversity may be the result of both freshwater and marine intrusions as illustrated by the ubiquitous distribution of estuarine sequences in the phylogenetic tree (Fig. 1). Overall, the high diversity in sediments may be explained by the heterogeneity of these habitats, which offer a large variety of potential niches for ammonia oxidation. In contrast, in the central Black Sea water, only one thaumarchaeotal subcluster was detected [51]. This agrees with the low PD value observed for marine planktonic *amoA* sequences, which was the result of closely related phylotypes rather than different lineage as illustrated by the low PSV value (Fig. 2b) and the concentration of marine planktonic sequences in one monophyletic cluster (Fig. 1). In the case of freshwater systems (i.e. both sediment and water column), the low PD value may result from lower sampling efforts. Indeed, inland water systems represent heterogeneous ecosystems and were identified as one of the largest reservoir of archaeal diversity [20]. However, freshwater ecosystems are by far less thoroughly sampled than marine habitats [24] and sampling is biased toward oligotrophic systems [24]–[26], [41]. Hence more research is called to describe freshwater AOA diversity.

**Figure 4:**
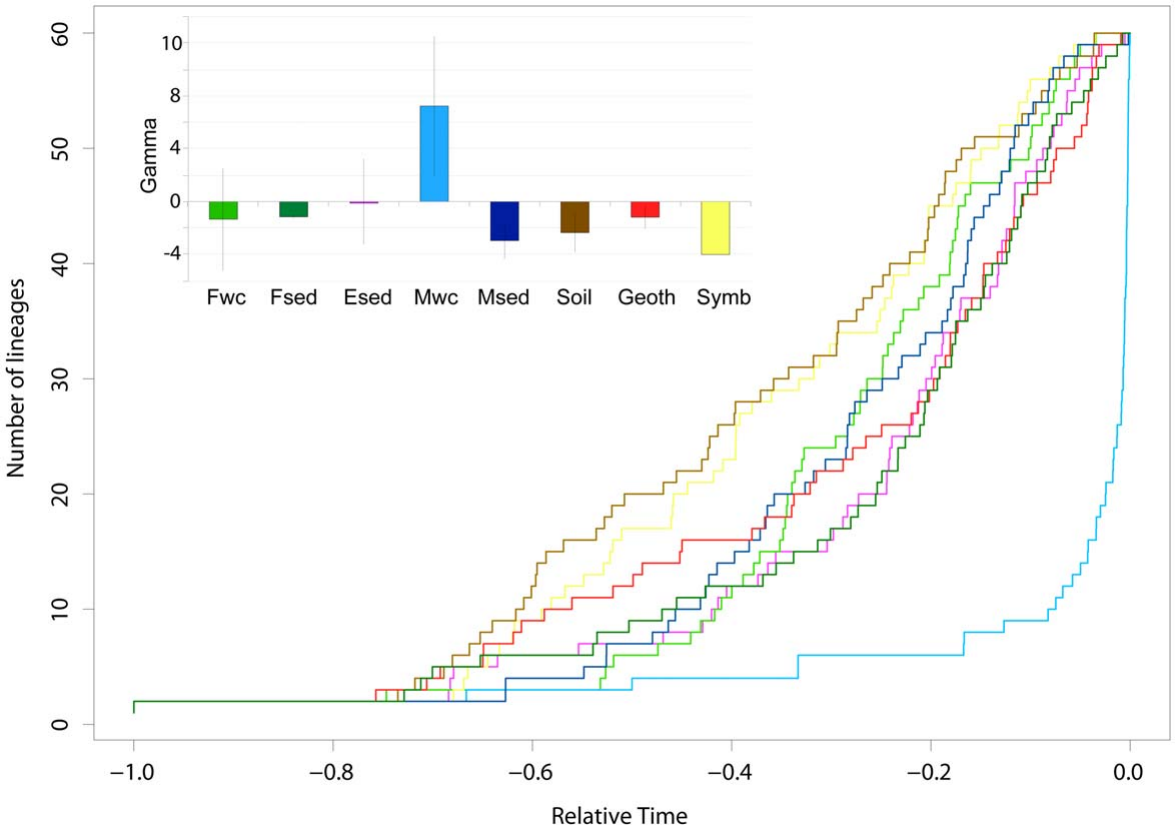
Diversification rates plotted as lineage through-time (ltt) plots based on ultrametric trees (penalized likelihood method). Bar plot in the upper left corner indicates the values γ (i.e. rate of cladogenesis) for each habitat.

The marked differences in AOA phylogenetic diversity and community structure among different habitats raised the question of the evolutionary processes underlying these patterns. One apparent reason may be the existence of distinct rates of cladogenesis over time among habitats. As phylogenies derived from molecular data, phylogenetic trees provide an indirect record of speciation events [52], the *amoA* phylogeny inferred in this study can be used to test this hypothesis. Accumulation of lineages as a function of a relative scale of time (ltt plot) were plotted for each habitat in order to assess departure from a constant rate of cladogenesis (i.e. γ = 0). Except for the marine water column, very similar ltt plots were observed for all habitats with a constant accumulation of lineages initiated closer to the root than to the tips of the tree were observed (Fig. 4). This resulted in constant diversification rates for the estuarine sediment and freshwater habitats and decelerating rates for the remaining habitats. These results must be taken cautiously as microbial evolutionary inferences suffer from limitations such as the lack of fossil records and the unknown range of microbial diversity [53]. The latter aspect is critical since the gamma statistical value calculated with the method developed by Pybus and Harvey [54] results in increasingly negative gamma values as the fraction of the sampled diversity decreases [55]. Hence, it is possible that the negative gamma values obtained would follow the general pattern for microorganisms assuming a constant diversification rate [55] if an exhaustive sampling of *amoA* diversity could be made. Departure from this general constant diversification pattern (i.e. acceleration or deceleration) has been observed previously in bacteria [56], archaea [57] and denitrifiers [21]. Very recently, it has been shown that the whole AOA community exhibited two fast diversification events separated by a long steady-state episode [22]. Interestingly, in the present study, only one habitat, the marine water column habitat, differed significantly from the general constant diversification pattern and displayed a recent diversification marked by an increase in the rate of cladogenesis (i.e. γ = 7.2±5.1) toward present time (Fig. 4). Discrepancies in the rate of cladogenesis and diversification patterns between both studies may rely in the methods used to calculate them. Here, we used a maximum likelihood method assuming a molecular clock since it provides a more reliable estimate of diversification than non-molecular clock methods [55]. The recent diversification of marine planktonic *amoA* sequences is consistent with the low PD and PSV values observed for this habitat. The factors resulting in an acceleration of the cladogenesis rate cannot be identified in this work but they may lie in the ecological context of speciation and extinction within marine planktonic systems.

Overall, our analysis provided further insight into the possible evolutionary mechanisms and environmental parameters that shape AOA community assembly at global scale. We unraveled a well-defined trend of community similarity by habitat type with salinity, temperature and lifestyle emerging as important environmental factors governing community phylogenetic similarity. Focusing on two aspects of macroevolution (i.e. the rate of cladogenesis over time and whether different habitats exhibit different diversification rates), we showed that planktonic marine habitats departed from other habitats by displaying a recent and accelerating diversification that may explain the low diversity of AOA in this ecosystem. Nonetheless, the lack of knowledge on true AOA diversification within certain habitats prevented definitive conclusions on the macroevolutionnary processes related to AOA diversification at the global scale.

## Materials and Methods

### Dataset constructions

The published literatures and GenBank database (before April, 2011) were surveyed to extract partial *amoA* gene sequences matching the following criteria: high-quality sequences without nucleotide ambiguities and with a length longer than 400 bp. Most sequences were amplified with the same primer set (Arch-amoAF: 5′-STAATGGTCTGGCTTAGACG-3′ and Arch-amoAR: 5′-GCGGCCATCCATCTGTATGT-3′) [45]. Variation in sampling efforts and methodologies among studies were homogenized by clustering *amoA* sequences at a 95% identity threshold using the MOTHUR software [58]. An AOA database of 1476 archaeal *amoA* sequences from 85 clone libraries globally distributed was assembled (see the Table S1).

Simultaneously, these clone libraries were classified into eight distinct habitats so as to discuss the preferred environment for AOA: soil, freshwater, freshwater sediment, estuary, marine water, marine sediment, geothermal systems and symbionts. The environmental factors were coded for every clone library using one semi-quantitative matrix on the basis of the gradients present in the eight distinct habitats: temperature (psychrophile to thermophile), salinity (hypersaline brines to freshwater), life style (plankton, soil, sediment and endosymbiont), trophic state (hypertrophic to oligotrophic) and oxygen concentrations (anoxic to oxic) (see Table S1).

### Phylogenetic analysis and diversity indices

*AmoA* gene sequences were aligned using the software MAFFT [59]. Poorly aligned positions and divergent regions of the DNA alignment were removed using the Gbloks software [60] resulting in 572 bp length fragments for the final analysis. Phylogenetic inference was carried out with RAxML version 7.2.8 [61] that estimates large phylogenies by maximum likelihood. The best phylogenetic tree estimated by the GTRCAT model with 1000 bootstrap replicates was drawn with iTOL [62].

Distance matrices based on relatedness between communities were calculated with Fast UniFrac [63]. Principal coordinate analysis (PCoA) plots were used to represent the ordering relationships obtained from the UniFrac distance matrices. In addition, a hierarchical clustering analysis (UPGMA algorithm with Jackknife supporting values) was run.

To determine the community similarity between the eight habitats delineated in this study, phylogenetic diversity (PD) indices for the eight habitats were calculated based on the summation of the branch length calculated from the *amoA* gene sequences within each habitat type [64]. To correct for unequal number of sequences, we calculated the mean PD of 1,000 randomized subsamples of each habitat [65]. The phylogenetic structure was evaluated with the phylogenetic species variability (PSV) index for each habitat [66]. PSV estimates phylogenetic diversity as the variance of a trait evolving under a neutral model. The PSV value changes toward 1 if species in a sample are unrelated, and their correlation is low indicating higher diversity in the sample as the species in a sample tend to be independent from each other. On the contrast, PSV value approaches to 0 if species are more related [66]. All these analyses were executed with the R package *picante* [67].

To compare the phylogenetic diversity between different habitats, a genetic distance matrix of the sequences from each habitat was made. This matrix was used in MOTHUR to calculate rarefaction curves [58].

### Diversification analysis

Diversification analyses were run on the habitats defined in this study. Because diversification analysis is sensible to sequence numbers, sampling efforts for each habitat were normalized by random resampling using the *sub-sample* function of MOTHUR [58]. Resampling was conducted ten times on each habitat and an equivalent number of ML rooted trees were constructed using the workflow described in the above section. These trees were rendered ultrametric (i.e. all branch tips are equidistant from the root) using the Sanderson's semi–parametric penalized likelihood approach [68] with the *chronopl* function of the ape package in R [69]. Several smoothing parameter (i.e. λ = 0; 0,5; 1) were tested in order to compare the results. However, no significant change in the rates of cladogenesis was observed. Visualization of diversification patterns was achieved by plotting the increasing number of lineages from the root nodes to the tips of the trees in a ltt plot (linear through time plot). For examining the rate of cladogenesis over time, we calculated the γ-statistic developed by Pybus and Harvey [54]. If diversification has been constant through time, the parameter γ = 0. If the diversification rate is slow, then γ<0, while γ>0 indicates acceleration in the rate of lineage accumulation [55]. We tested whether there was a difference between habitat cladogenesis rates using a Kruskal-Wallis test on the γ values.

All these analyses were run in R software (http://www.r-project.org/) with the package *ape* [69].

## Supporting Information

Table S1 **Summary of the 85 archaeal libraries included in the analysis and the environmental matrix associated.**
(DOCX)

Figure S1 **Rarefaction curves of archaeal** ***amoA*** **gene sequences retrieved from all the references on the basis of 5% distance cut-off calculated from MOTHUR software.**
(TIF)
