# Supplementary material for: Global Ecological Pattern of Ammonia-Oxidizing Archaea
Source: PLoS One. 2013 Feb 28;8(2):e52853. doi: 10.1371/journal.pone.0052853 (PMC3585293; doi:10.1371/journal.pone.0052853)
Supplement: Table S1 — Summary of the 85 archaeal libraries included in the analysis and the environmental matrix associated. (DOCX) [file pone.0052853.s001.docx]

**Table S1 Summary of the 85 archaeal libraries included in the analysis and the environmental matrix associated**.

| Environment | Site location | OUTs at 95% (total sequences) | Number of Clones  libraries | Study | T. | Life  style | Salinity | Oxygen | Trophic  State (N) |
| --- | --- | --- | --- | --- | --- | --- | --- | --- | --- |
| Freshwater | Qinghai Lake | 4(10) | 5 | Jiang et al. 2009 | 2 | 1 | 3 | 0 | 1 |
|  | Lake Kivu, Congo | 1(16) | 1 | Llirós et al., 2010 | 2 | 1 | 4 | 0 | 1 |
|  | Dongjiang River, China | 6(119) | 4 | Liu et al., 2011 | 2 | 1 | 4 | 0 | 1 |
|  | Alpine lakes/winter | 17(100) | 1 | Auguet et al., 2011 | 1 | 1 | 4 | 0 | 1 |
|  | Alpine lakes/spring | 12(12) | 1 | Auguet et al., 2011 | 1 | 1 | 4 | 0 | 1 |
|  | Alpine lakes/summer | 24(29) | 1 | Auguet et al., 2011 | 2 | 1 | 4 | 0 | 1 |
|  | Macrophate, China/water(c3) | 2(30) | 1 | Wei et al., 2011 | 2 | 1 | 4 | 0 | 3 |
| Freshwater  sediment | Wetland, China | 8(11) | 1 | Wang et al., 2011 | 2 | 2 | 4 | 0 | 2 |
|  | Wetland, China/Freshwater marsh | 9(10) | 1 | Wang et al., 2011 | 2 | 2 | 4 | 0 | 2 |
|  | Wetland, China/Baiyangdian Lake | 10(11) | 1 | Wang et al., 2011 | 2 | 2 | 4 | 0 | 2 |
|  | Wetland, China/Paddy field | 6(20) | 1 | Wang et al., 2011 | 2 | 2 | 4 | 0 | 3 |
|  | Qinghai Lake/salinity | 17(44) | 6 | Jiang et al. 2009 | 1 | 2 | 3 | 1 | 1 |
|  | Lake Taihu, China | 12(99) | 6 | Wu et al. 2010 | 2 | 2 | 4 | 0 | 3 |
|  | Macrophytes-dystrophic | 4(32) | 4 | Herrmann et al. 2009 | 2 | 2 | 4 | 0 | 2 |
|  | Macrophytes-oligotrophic | 10(62) | 5 | Herrmann et al. 2009 | 2 | 2 | 4 | 0 | 1 |
|  | Macrophytes-mesotrophic | 12(69) | 4 | Herrmann et al. 2008; 2009 | 2 | 2 | 4 | 0 | 2 |
|  | Macrophytes-root | 13(80) | 9 | Herrmann et al. 2009 | 2 | 2 | 4 | 0 | 2 |
|  | Macrophate, China/sediments (n) | 2(30) | 1 | Wei et al., 2011 | 2 | 1 | 4 | 0 | 3 |
|  | Macrophate, China/root | 12(88) | 3 | Wei et al., 2011 | 2 | 1 | 4 | 0 | 3 |
| Soil (pristine/  Agricultural/  amended) | Soils/sandy ecosystem soil/6070 | 8(36) | 1 | Leininger et al., 2006 | 2 | 3 | 4 | 0 | 1 |
|  | Soils/sandy ecosystem soil/010 | 6(36) | 1 | Leininger et al., 2006 | 2 | 3 | 4 | 0 | 1 |
|  | Melton Branch Watershed Oak Ridge,TN/Cu | 2(29) | 1 | Hansel et al. 2008 | 2 | 3 | 4 | 0 | 1 |
|  | Melton Branch Watershed Oak Ridge,TN/CS | 15(49) | 2 | Francis et al. 2005;  Hansel et al. 2008 | 2 | 3 | 4 | 0 | 1 |
|  | Upland red soil, China | 19(20) | 1 | He et al., 2007 | 2 | 3 | 4 | 0 | 2 |
|  | Mount Everest (Tibetan Plateau)/M1-M3 | 13(23) | 3 | Zhang et al. 2009 | 1 | 3 | 4 | 1 | 2 |
|  | Mount Everest (Tibetan Plateau)M4-M12 | 17(46) | 9 | Zhang et al. 2009 | 1 | 3 | 4 | 1 | 1 |
|  | Soil under tree, Oregen/coastal andisols | 9(18) | 1 | Boyle-Yarwood et al. 2008 | 2 | 3 | 4 | 0 | 1 |
|  | Soil with pH gradient(7.5/4.9) | 11(68) | 2 | Nicol et al., 2008 | 2 | 3 | 4 | 0 | 2 |
|  | Agricultural soil, Scotland/Temperature | 5(23) | 6 | Tourna et al., 2008 | 2 | 3 | 4 | 0 | 2 |
|  | Paddy rhizosphere soil | 14(23) | 2 | Chen et al. 2008 | 2 | 3 | 4 | 0 | 2 |
|  | Barley-planted soil | 25(124) | 1 | Glaser et al., 2010 | 2 | 3 | 4 | 0 | 1 |
|  | Peat wetland soil/upper | 6(46) | 3 | Höfferle et al., 2010 | 2 | 3 | 4 | 0 | 1 |
|  | Peat wetland soil/lower | 7(41) | 3 | Höfferle et al., 2010 | 2 | 3 | 4 | 1 | 2 |
| Marine  sediment | Salt marsh sediments | 19(125) | 3 | Moin et al. 2009 | 2 | 2 | 1 | 0 | 2 |
|  | Korean marine sediment (over 500m) | 31(207) | 4 | Park et al. 2008 | 1 | 2 | 1 | 1 | 1 |
|  | West Pacific Continental Margin/deep sea | 84(167) | 8 | Dang et al. 2009 | 1 | 2 | 1 | 1 | 1 |
|  | Northeastern Japan Sea/CR+CBK/deep sea | 6(20) | 2 | Nakagawa et al., 2007 | 1 | 2 | 1 | 1 | 1 |
|  | Okhotsk Sea/deep sea | 31(68) | 8 | Dang et al. 2010 | 1 | 2 | 1 | 1 | 1 |
|  | South China Sea/shallow | 103(116) | 7 | Cao et al., 2011 | 2 | 2 | 1 | 0 | 1 |
|  | South China Sea/deep | 75(86) | 6 | Cao et al., 2011 | 1 | 2 | 1 | 1 | 1 |
| Marine water  column | Black sea (15.7, 15.8, 15.9) | 4(64) | 3 | Francis et al. 2005 | 2 | 1 | 1 | 1 | 2 |
|  | Black Sea (mRNA) | 9(40) | 5 | Lam et al. 2007;  Labrenz et al. 2010 | 1 | 1 | 1 | 1 | 1 |
|  | Eastern Tropical North Pacific (ETNP) | 11(26) | 1 | Francis et al. 2005 | 2 | 1 | 2 | 1 | 1 |
|  | Monterey Bay | 11(50) | 2 | Francis et al., 2005;  Mincer et al., 2007 | 2 | 1 | 1 | 0 | 1 |
|  | Northeastern Japan Sea/NR+NY | 7(20) | 2 | Nakagawa et al., 2007 | 1 | 1 | 1 | 1 | 1 |
|  | North Pacific Gyre (ALOHA) | 12(52) | 5 | Mincer et al. 2007 | 1 | 1 | 1 | 1 | 1 |
|  | Deep North Atlantic | 18(64) | 3 | Agogué et al., 2008 | 1 | 1 | 2 | 1 | 1 |
|  | Gulf of California/coastal/60m+450m | 11(93) | 4 | Beman et al. 2008 | 2 | 1 | 1 | 0 | 1 |
|  | Arctic Ocean | 11(62) | 1 | Kalanetra et al. 2009 | 1 | 1 | 1 | 0 | 1 |
|  | Antarctic coastal waters | 9(31) | 1 | Kalanetra et al. 2009 | 1 | 1 | 1 | 0 | 1 |
|  | Central California Current/cDNA | 42(51) | 6 | Santoro et al. 2010 | 2 | 1 | 1 | 0 | 1 |
|  | Eastern South Pacific/oxygen deficient | 27(105) | 8 | Molina et al. 2010 | 2 | 1 | 1 | 1 | 1 |
|  | Tyrrhenian deep waters/DNA+mRNA | 6(25) | 4 | Yakimov et al., 2008; 2011 | 1 | 1 | 1 | 1 | 1 |
|  | Coastal Arctic ocean | 4 (44) | 2 | Christman et al., 2011 | 1 | 1 | 1 | 0 | 1 |
| Geothermal system : Hydrothermal  Vent/hot springs | Geothermal mine adit/mat | 5(82) | 8 | Spear et al. 2007 | 3 | 2 | 4 | 1 | 1 |
|  | Hot springs in Ice land and Kamchatka | 5(38) | 11 | Reigstad et al. 2008 | 3 | 2 | 4 | 0 | 1 |
|  | Global hotsprings/Kamchatka | 34(392) | 3 | Zhang et al. 2008 | 3 | 2 | 4 | 1 | 2 |
|  | Global hotsprings/China, Tengchong | 40(111) | 5 | Zhang et al. 2008 | 3 | 2 | 4 | 1 | 2 |
|  | Global hotsprings/Great Basin | 25(49) | 7 | Zhang et al. 2008 | 3 | 2 | 4 | 1 | 1 |
|  | Global hotsprings/Yellowstone NP | 22(40) | 6 | Zhang et al. 2008 | 3 | 2 | 4 | 1 | 2 |
|  | Thermal spring in Austrian Central Alps | 4(4) | 1 | Weidler et al. 2007 | 3 | 1 | 4 | 0 | 1 |
|  | Juan de fuca ridge | 9(93) | 3 | Wang et al. 2009 | 3 | 2 | 1 | 1 | 1 |
|  | Southern Mariana Trough | 8(10) | 2 | Kato et al., 2009 | 3 | 2 | 1 | 1 | 1 |
|  | Yunnan Province, China | 40(81) | 11 | Zhang et al. 2008;  Jiang et al. 2010 | 3 | 2 | 4 | 0 | 1 |
|  | Southern Okinawa Trough(763S+816W) | 12(14) | 2 | Nunoura et al., 2010 | 3 | 2 | 1 | 1 | 1 |
|  | Kamchatka Hot Springs | 32(379) | 8 | Zhao et al., 2011 | 3 | 2 | 4 | 1 | 1 |
| Estuarine sediments/salt marsh | Elkhorn Slough estuary | 68(125) | 4 | Wankel et al., 2011 | 2 | 2 | 1 | 0 | 3 |
|  | Bahia del Tobari, Mexico | 43(218) | 10 | Beman & Francis, 2006 | 2 | 2 | 1 | 0 | 2 |
|  | Subterranean estuary/ Huntington Beach | 22(84) | 2 | Santoro et al., 2008 | 2 | 2 | 1 | 0 | 1 |
|  | Subterranean estuary/ Huntington Beach | 40(231) | 4 | Santoro et al., 2008 | 2 | 2 | 3 | 0 | 1 |
|  | Changjiang Estuary, China | 29(47) | 3 | Dang et al., 2008 | 2 | 2 | 1 | 0 | 2 |
|  | San Francisco Bay estuary/lower salinity | 19(132) | 7 | Mosier & Francis, 2008 | 2 | 2 | 3 | 0 | 2 |
|  | San Francisco Bay estuary/higher salinity | 54(282) | 11 | Mosier & Francis, 2008 | 2 | 2 | 2 | 0 | 2 |
|  | Fitzroy river estruay (613-615-619)/Sept. | 27(132) | 3 | Abell eta l., 2010 | 2 | 2 | 1 | 0 | 2 |
|  | Plum Island Sound estuary/P22 | 17(93) | 4 | Bernhard et al., 2010 | 2 | 2 | 3 | 0 | 2 |
|  | Plum Island Sound estuary/P14 | 26(212) | 4 | Bernhard et al., 2010 | 2 | 2 | 2 | 0 | 2 |
|  | Plum Island Sound estuary/R8C | 24(147) | 4 | Bernhard et al., 2010 | 2 | 2 | 1 | 0 | 2 |
|  | Douro River estuary, Portugal | 17(40) | 1 | Magalhães et al., 2009 | 2 | 2 | 5 | 0 | 2 |
|  | Pearl River Estuary | 29(100) | 1 | Jin et al., 2011 | 2 | 2 | 2 | 0 | 2 |
|  | Mai Po wetland | 14(193) | 6 | Cao et al., 2011 | 2 | 2 | 2 | 0 | 2 |
| symbionts | Corals | 33(210) | 7 | Beman et al., 2007 | 2 | 4 | 6 | 0 | 2 |
|  | Marine sponges | 15(26) | 12 | Steger et al., 2008 | 2 | 4 | 6 | 0 | 2 |
|  | Sponge *Aplysina aerophoba* | 2(10) | 1 | Bayer et al., 2008 | 2 | 4 | 6 | 0 | 2 |
|  | Sponge *Geodia barretti* | 1(38) | 1 | Hoffmann et al., 2009 | 1 | 4 | 6 | 0 | 2 |
|  | Sponge *Xestospongia muta* | 8(109) | 7 | López-Legentil et al., 2010 | 2 | 4 | 6 | 0 | 2 |

Temperature: 1 = psychrophile, 2 = mesophile, 3 = thermophile, Life style: 1 = water, 2 = sediment, 3 = soil, 4= endosymbiont; Salinity: 1 = saline 2 = middle-salinity 3 = low-salinity, 4 = non-saline, 5 = mixture, 6 = endosymbiont; Oxygene: 0 = oxic, 1 = anoxic; Trophic state: 1 = oligotrophic, 2 = mesotrophic, 3 = hypertrophic.

**References for supplementary Table S1.**

1. Llirós M, Gich F, Plasencia A, Auguet JC, Darchambeau F, et al. (2010) Vertical distribution of ammonia-oxidizing crenarchaeota and methanogens in the epipelagic waters of Lake Kivu (Rwanda-Democratic Republic of the Congo). Appl Environ Microbiol 76: 6853-6863.
2. Jiang, H, Dong, H, Yu, B, Lv, G, Deng, S, et al. (2009) Diversity and Abundance of Ammonia-Oxidizing Archaea and Bacteria in Qinghai Lake, Northwestern China Geomicrobiology J 26: 199-211.
3. Liu Z, Huang S, Sun G, Xu Z, Xu M (2011) Diversity and abundance of ammonia-oxidizing archaea in the Dongjiang River, China. Microbiol Res 166: 337-345.
4. Auguet JC, Nomokonova N, Camarero L, Casamayor EO (2011) Seasonal changes of freshwater ammonia-oxidizing archaeal assemblages and nitrogen species in oligotrophic alpine lakes. Appl Environ Microbiol 77:1937-1945.
5. Wei B, Yu X, Zhang S, Gu L (2011) Comparison of the community structures of ammonia-oxidizing bacteria and archaea in rhizoplanes of floating aquatic macrophytes. Microbiol Res 166: 468-474.
6. Wang S, Wang Y, Feng X, Zhai L, Zhu G (2011) Quantitative analyses of ammonia-oxidizing Archaea and bacteria in the sediments of four nitrogen-rich wetlands in China. Appl Microbiol Biotechnol 90: 779-787.
7. Wu Y, Xiang Y, Wang J, Zhong J, He J, et al. (2010) Heterogeneity of archaeal and bacterial ammonia-oxidizing communities in Lake Taihu, China. Environ Microbiol Reports 2: 569-576.
8. Herrmann M, Saunders AM, Schramm A (2008) Archaea dominate the ammonia-oxidizing community in the rhizosphere of the freshwater macrophyte Littorella uniflora. Appl Environ Microbiol 74: 3279-3283.
9. Herrmann M, Saunders AM, Schramm A (2009) Effect of lake trophic status and rooted macrophytes on community composition and abundance of ammonia-oxidizing prokaryotes in freshwater sediments. Appl Environ Microbiol 75: 3127-3136.
10. Leininger S, Urich T, Schloter M, Schwark L, Qi J, et al. (2006) Archaea predominate among ammonia-oxidizing prokaryotes in soils. Nature 442:806-809.
11. Hansel CM, Fendorf S, Jardine PM, Francis CA (2008) Changes in bacterial and archaeal community structure and functional diversity along a geochemically variable soil profile. Appl Environ Microbiol 74: 1620-1633.
12. He JZ, Shen JP, Zhang LM, Zhu YG, Zheng YM, et al. (2007) Quantitative analyses of the abundance and composition of ammonia-oxidizing bacteria and ammonia-oxidizing archaea of a Chinese upland red soil under long-term fertilization practices. Environ Microbiol 9: 2364-2374.
13. Zhang LM, Wang M, Prosser JI, Zheng YM, He JZ (2009) Altitude ammonia-oxidizing bacteria and archaea in soils of Mount Everest. FEMS Microbiol Ecol 70: 52-61.
14. Boyle-Yarwood SA, Bottomley PJ, Myrold DD (2008) Community composition of ammonia-oxidizing bacteria and archaea in soils under stands of red alder and Douglas fir in Oregon. Environ Microbiol 10: 2956-2965.
15. Nicol GW, Leininger S, Schleper C, Prosser JI (2008) The influence of soil pH on the diversity, abundance and transcriptional activity of ammonia oxidizing archaea and bacteria. Environ Microbiol 10: 2966-2978.
16. Tourna M, Freitag TE, Nicol GW, Prosser JI (2008) Growth, activity and temperature responses of ammonia-oxidizing archaea and bacteria in soil microcosms. Environ Microbiol 10: 1357-1364.
17. Chen XP, Zhu YG, Xia Y, Shen JP, He JZ (2008) Ammonia-oxidizing archaea: important players in paddy rhizosphere soil? Environ Microbiol 10: 1978-1987.
18. Höfferle Š, Nicol GW, Pal L, Hacin J, Prosser JI, et al. (2010) Ammonium supply rate influences archaeal and bacterial ammonia oxidizers in a wetland soil vertical profile. FEMS Microbiol Ecol 74: 302-315.
19. Moin NS, Nelson KA, Bush A, Bernhard AE (2009) Distribution and diversity of archaeal and bacterial ammonia oxidizers in salt marsh sediments. Appl Environ Microbiol 75:7461-7468.
20. Park BJ, Rhee SK (2008) Comparative analysis of archaeal 16S rRNA and *amoA* genes to estimate the abundance and diversity of ammonia-oxidizing archaea in marine sediments. Extremophiles 12: 605-615.
21. Dang, H., Li, J., Zhang, X., Li, T., Tian, F. & Jin, W. (2009a). Diversity and spatial distribution of *amoA*-encoding archaea in the deep-sea sediments of the tropical West Pacific Continental Margin. *Journal of Applied Microbiology, 106,* 1482-1493.
22. Nakagawa T, Mori K, Kato C, Takahashi R, Tokuyama T (2007) Distribution of cold-adapted ammonia-oxidizing microorganisms in the deep-ocean of the northeastern Japan Sea. Microbes Environ 22: 365-372.
23. Dang H, Luan XW, Chen R, Zhang X, Guo L, et al. (2010) Diversity, abundance and distribution of *amoA*-encoding archaea in deep-sea methane seep sediments of the Okhotsk Sea. FEMS Microbiol Ecol 72: 370-385.
24. Cao H, Hong Y, Li M, Gu JD (2011) Diversity and Abundance of Ammonia-oxidizing Prokaryotes in Sediments from the Coastal Margin to the South China Sea. Antonie van Leeuwenhoek DOI: 10.1007/s10482-011-9610-1.
25. Cao H, Li M, Hong Y, Gu JD (2011) Phylogenetic Diversity and Ecological Pattern of Ammonia-oxidizing Archaea in the Surface Sediments of the Western Pacific. Microbiol Ecol DOI 10.1007/s00248-011-9901-0.
26. Francis CA, Roberts KJ, Beman JM, Santoro AE, Oakley BB (2005) Ubiquity and diversity of ammonia-oxidizing archaea in water columns and sediments of the ocean. Proc Natl Acad Sci USA 102: 14683-14688.
27. Lam P, Jensen MM, Lavik G, McGinnis DF, Muller B, et al. (2007) Linking crenarchaeal and bacterial nitrification to anammox in the Black Sea. Proc Natl Acad Sci USA 104: 7104-7109.
28. Labrenz M, Sintes E, Toetzke F, Zumsteg A, Herndl GJ, et al. (2010) Relevance of a crenarchaeotal subcluster related to *Candidatus* Nitrosopumilus maritimus to ammonia oxidation in the suboxic zone of the central Baltic Sea. ISME J 4: 1496-1508.
29. Mincer TJ, Church MJ, Taylor LT, Preston C, Karl DM, et al. (2007) Quantitative distribution of presumptive archaeal and bacterial nitrifiers in Monterey Bay and the North Pacific Subtropical Gyre. Environ Microbiol 9:1162-1175.
30. Agogué H, Brink M, Dinasquet J, Herndl GJ (2008) Major gradients in putatively nitrifying and non-nitrifying Archaea in the deep North Atlantic. Nature 456:788-791.
31. Beman JM, Popp BN, Francis CA (2008) Molecular and biogeochemical evidence for ammonia oxidation by marine *Crenarchaeota* in the Gulf of California. ISME J 2: 429-441.
32. Kalanetra KM, Bano N, Hollibaugh JT (2009) Ammonia-oxidizing Archaea in the Arctic Ocean and Antarctic coastal waters. Environ Microbiol 11: 2434-2445.
33. Santoro AE, Casciotti KL, Francis CA (2010) Activity, abundance and diversity of nitrifying archaea and bacteria in the central California Current. Environ Microbiol 12: 1989-2006.
34. Molina V, Belmar L, Ulloa O (2010) High diversity of ammonia-oxidizing archaea in permanent and seasonal oxygen-deficient waters of the eastern South Pacific. Environ Microbiol 12: 2450-2465.
35. Yakimov, M.M., Conoa, V.L., and Denaroa, R. (2009) A first insight into the occurrence and expression of functional amoA and accA genes of autotrophic and ammonia-oxidizing bathypelagic Crenarchaeota of Tyrrhenian Sea *Deep-Sea Res Pt II* **56**: 748-754.
36. Yakimov MM, Cono VL, Smedile F, Deluca TH, Juárez S, et al. (2011) Contribution of crenarchaeal autotrophic ammonia oxidizers to the dark primary production in Tyrrhenian deep waters (Central Mediterranean Sea). ISME J 5: 945-961.
37. Christman GD, Cottrell MT, Popp BN, Gier E, Kirchman DL (2011) Abundance, diversity, and activity of ammonia-oxidizing prokaryotes in the coastal Arctic ocean in summer and winter. Appl Environ Microbiol 77:2026-2034.
38. Spear JR, Barton HA, Robertson CE, Francis CA, Pace NR (2007) Microbial community biofabrics in a geothermal mine adit. Appl Environ Microbiol 73: 6172-6180.
39. Reigstad, L.J., Richter, A., Daims, H., Urich, T., Schwark, L., & Schleper, C. (2008). Nitrification in terrestrial hot springs of Iceland and Kamchatka. *FEMS Microbiology Ecology, 64,* 167-174.
40. Zhang CL, Ye Q, Huang Z, Li W, Chen J, et al. (2008) Global occurrence of archaeal *amoA* genes in terrestrial hot springs. Appl Environ Microbiol 74: 6417-6426.
41. Weidler GW, Dornmayr-Pfaffenhuemer M, Gerbl FW, Heinen W, Stan-Lotter H (2007) Communities of archaea and bacteria in a subsurface radioactive thermal spring in the Austrian Central Alps, and evidence of ammonia-oxidizing *Crenarchaeota*. Appl Environ Microbiol 73: 259-270.
42. Wang S, Xiao X, Jiang L, Peng X, Zhou H, et al. (2009) Diversity and abundance of ammonia-oxidizing archaea in hydrothermal vent chimneys, Juan de Fuca Ridge. Appl. Environ. Microbiol 75: 4216-4220.
43. Kato S, Kobayashi C, Kakegawa T, Yamagishi A (2009) Microbial communities in iron-silica-rich microbial mats at deep-sea hydrothermal fields of the Southern Mariana Trough. Environ Microbiol 11: 2094-2111.
44. Jiang H, Huang Q, Dong H, Wang P, Wang F, et al. (2010) RNA-based investigation of ammonia-oxidizing archaea in hot springs of Yunnan Province, China. Appl Environ Microbiol 76: 4538-4541.
45. Nunoura T, Oida H, Nakaseama M, Kosaka A, Ohkubo SB, et al. (2010) Archaeal diversity and distribution along thermal and geochemical gradients in hydrothermal sediments at the Yonaguni Knoll IV hydrothermal field in the Southern Okinawa trough. Appl Environ Microbiol 76: 1198-11211.
46. Zhao W, Song Z, Jiang H, Li W, Mou X, et al. (2011) Ammonia-oxidizing archaea in Kamchatka hot springs. Geomicrobiol J 28: 149-151.
47. Wankel SD, Mosier AC, Hansel CM, Paytan A, Francis CA (2011) Spatial Variability in Nitrification Rates and Ammonia-Oxidizing Microbial Communities in the Agriculturally-impacted Elkhorn Slough Estuary. Appl Environ Microbiol 77: 269-280.
48. Beman JM, Francis CA (2006) Diversity of ammonia-oxidizing archaea and bacteria in the sediments of a hypernutrified subtropical estuary: Bahia del Tobari, Mexico. Appl Environ Microbiol 72: 7767-7777.
49. Santoro AE, Francis CA, de Sieyes NR, Boehm AB (2008) Shifts in the relative abundance of ammonia-oxidizing bacteria and archaea across physicochemical gradients in a subterranean estuary. Environ Microbiol 10: 1068-1079.
50. Dang H, Zhang X, Sun J, Li T, Zhang Z, et al. (2008) Diversity and spatial distribution of sediment ammonia-oxidizing crenarchaeota in response to estuarine and environmental gradients in the Changjiang Estuary and East China Sea. Microbiology 154: 2084-2095.
51. Mosier AC, Francis CA (2008) Relative abundance and diversity of ammonia-oxidizing archaea and bacteria in the San Francisco Bay estuary. Environ Microbiol 10: 3002-3016.
52. Abell GC, Banks J, Ross DJ, Keane JP, Robert SS, et al. (2011) Effects of estuarine sediment hypoxia on nitrogen fluxes and ammonia oxidizer gene transcription. FEMS Microbiol Ecol 75:111-122.
53. Bernhard AE, Landry ZC, Blevins A, de la Torre JR, Giblin AE, et al. (2010) Abundance of ammonia-oxidizing archaea and bacteria along an estuarine salinity gradient in relation to potential nitrification rates. Appl Environ Microbiol 76:1285-1289.
54. Magalhães CM, Machado A, Bordalo AA (2009) Temporal variability in the abundance of ammonia-oxidizing bacteria vs. archaea in sandy sediments of the Douro River estuary, Portugal. Aquat Microb Ecol 56:13-23
55. Jin T, Zhang T, Ye L, Lee OO, Wong YH, et al. (2011) Diversity and quantity of ammonia-oxidizing Archaea and Bacteria in sediment of the Pearl River Estuary, China. Appl Microbiol Biotechnol 90:1137-1145.
56. Beman JM, Roberts KJ, Wegley L, Rohwer F, Francis CA (2007) Distribution and diversity of archaeal ammonia monooxygenase genes associated with corals. Appl Environ Microbiol 73:5642-5647.
57. Steger D, Ettinger-Epstein P, Whalan S, Hentschel U, de Nys R, et al. (2008) Diversity and mode of transmission of ammonia-oxidizing archaea in marine sponges. Environ Microbiol 10: 1087-1094.
58. Bayer K, Schmitt S, Hentschel U (2008) Physiology, phylogeny and in situ evidence for bacterial and archaeal nitrifiers in the marine sponge *Aplysina aerophoba*. Environ Microbiol 10: 2942-2955.
59. Hoffmann F, Radax R, Woebken D, Holtappels M., Lavik, G, et al. (2009) Complex nitrogen cycling in the sponge *Geodia barretti*. Environ Microbiol 11: 2228-2243.
60. López-Legentil S, Erwin PM, Pawlik JR, Song B (2010) Effects of sponge bleaching on ammonia-oxidizing Archaea: distribution and relative expression of ammonia monooxygenase genes associated with the barrel sponge *Xestospongia muta*. Microb Ecol 60: 561-571.
