# Supplementary figures and images for: Global Ecological Pattern of Ammonia-Oxidizing Archaea
Source: PLoS One. 2013 Feb 28;8(2):e52853. doi: 10.1371/journal.pone.0052853 (PMC3585293; doi:10.1371/journal.pone.0052853)

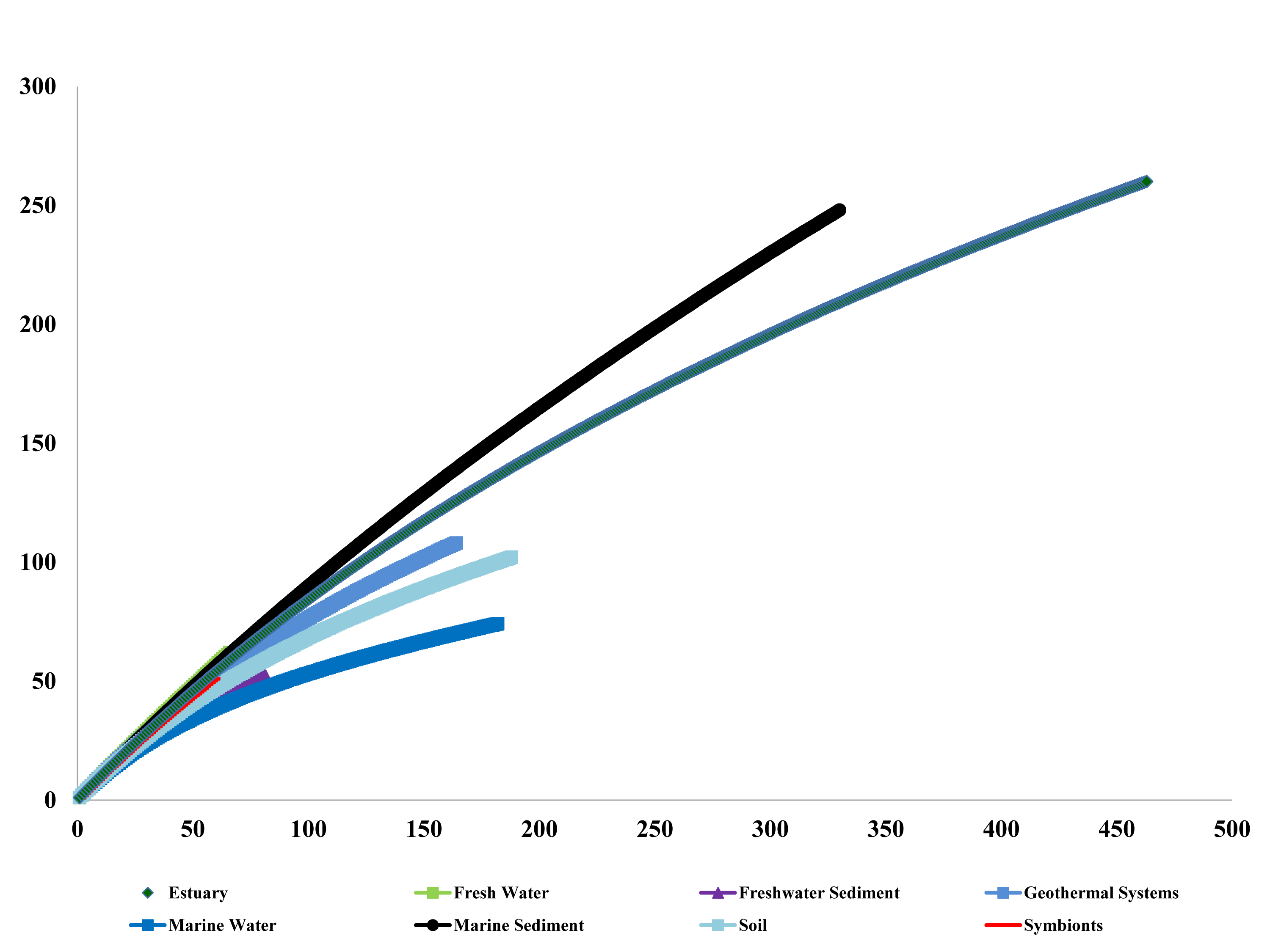

Supplement: Figure S1 — Rarefaction curves of archaeal amoA gene sequences retrieved from all the references on the basis of 5% distance cut-off calculated from MOTHUR software. (TIF) [file pone.0052853.s002.tif]
